# Endorsement of HIV-related stigma among men in Ghana: What are the determinants?

**DOI:** 10.1371/journal.pone.0305811

**Published:** 2024-07-01

**Authors:** Sulemana Ansumah Saaka, Roger Antabe

**Affiliations:** 1 Department of Geography and Environment, University of Western Ontario, London, Canada; 2 Department of Health and Society, University of Toronto Scarborough, Toronto, Canada; University of the Witwatersrand Johannesburg, SOUTH AFRICA

## Abstract

**Introduction:**

Stigma and discrimination against people living with HIV (PLHIV) remain a major barrier to effective HIV prevention. Despite the understanding that the creation of a socially inclusive environment for PLHIV is crucial for the promotion of testing, status disclosure, and treatment uptake, HIV stigma persists. Additionally, evidence suggests the endorsement of HIV stigma may be gender specific. Nonetheless, very little is known about the factors influencing men’s discrimination against PLHIV in the Ghanaian context. Guided by the theory of planned behavior, our study fills this void by exploring the factors associated with the endorsement of HIV stigma in Ghana.

**Methods:**

Utilizing a nationally representative data from the 2022 Ghana Demographic and Health Survey (DHS) (N = 7044 men with ages ranging from 15–49 years), and applying logistic regression models, this study examined the factors associated with the endorsement of HIV-related stigma in Ghana.

**Results:**

The notion that HIV can be transmitted through the sharing of food with PLHIV was significantly associated with increased odds of stigma endorsement against children with HIV (OR = 3.381; P<0.001) and vendors with HIV (OR = 3.00; P<0.001). On the contrary, knowing that a healthy-looking person can have HIV was significantly associated with decreased odds of endorsement of stigma against children living with HIV (OR = 0.505; P<0.001), and vendors living with HIV (OR = 0.573; P<0.001). Likewise, having knowledge of drugs that help PLHIV to live longer, was significantly associated with decreased odds of stigma endorsement against children living with HIV (OR = 0.768; P<0.001), and vendors living with HIV (OR = 0.719; P<0.001). Moreover, participants with higher educational attainment reported lower odds of stigma endorsement against children living with HIV (OR = 0.255; P<0.01), and vendors living with HIV (OR = 0.327; P<0.01). Furthermore, age was significant and inversely associated with the endorsement of HIV stigma against children living with HIV (OR = 0.951; P<0.05), and vendors living with HIV (OR = 0.961; P<0.05). Also, wealth, ethnicity, and the region of residence significantly predicted endorsement of HIV stigma.

**Conclusion:**

For Ghana to achieve UNAIDS target 95-95-95 by 2030, targeted educational campaigns are necessary to dispel misconceptions about HIV and to promote social inclusion for reducing HIV-related stigma and discrimination in the country.

## Introduction

Globally, stigma and discrimination against People living with HIV (PLHIV) remain a persistent barrier to effective HIV prevention. For people who are unaware of their HIV serostatus, HIV stigma and discrimination may work to prevent them from testing. Those who are aware of their HIV positive may decline to disclose their status or initiate timely treatment with further implications for their overall health and wellbeing [1–3]. A recent report by the Joint United Nations Programme on HIV/AIDS (UNAIDS) indicates that across all countries with available data, about 21% of PLHIV reported being denied health care in the past and are 2.4 times more likely to delay treatment until they are very ill due to the stigma and discrimination associated with HIV [4]. In contexts such as sub-Saharan Africa (SSA) where HIV is a major public health issue, the problem of HIV stigma has been noted as a major barrier to achieving targets such as the 95-95-95 set by UNAIDS [5]. For instance, a study by Sartorius et al. [6] revealed in a modelling simulation across 44 SSA countries that most countries in the region are falling short of HIV prevention targets, as HIV stigma works to deter people from testing, disclosing their statuses, or even adhering to treatment [7, 8]. Despite the explicit scientific knowledge on this globally important topic (i.e., HIV/AIDS), curated evidence suggests that persistent HIV misconceptions and gaps in HIV knowledge may be serving as a major driver of HIV stigma [9], even among healthcare providers [10–15]. In Ghana, the stigma index among PLHIV is 18.1% [16] which is relatively high due to lower levels of accurate HIV knowledge among the general population. For instance, the Demographic and Health Survey (DHS) report of Ghana indicated that even though general awareness about HIV/AIDS is high in Ghana, the majority of Ghanaian adults lack accurate knowledge of HIV transmission [17]. The report further revealed that only 18% and 30% of adult women and adult men in Ghana have comprehensive knowledge about HIV/AIDS, respectively. Using data from the 2003, 2008, and 2014 Ghana DHS datasets for trend analysis, Fenny et al. [11] further discovered that there has been a significant decrease in comprehensive knowledge on HIV/AIDS in Ghana from 72% in 2008 to 59% in 2013. Moreover, the 2022 DHS report of Ghana shows that only 36% of young women and 37% of young men aged 15–24 are knowledgeable about HIV prevention [18]. Meanwhile, lower levels of HIV knowledge is reportedly associated with elevated misconceptions about HIV transmission [19], consequently leading to discrimination attitudes towards PLHIV.

In addition to the lack of accurate knowledge about HIV, other studies have identified the fear of contracting HIV, social and moral perceptions about HIV and PLHIV, educational attainment, and geographical location as drivers of stigma and discrimination towards PLHIV [20, 21]. Refusal to share food, common spaces, items, or even hold/shake hands are examples of the everyday discriminatory behaviors faced by PLHIV, which adversely impact their social and psychological well-being [21]. Notwithstanding the fact that the health of PLHIV can be enhanced with anti-retroviral treatment (ART), they continue to face discrimination, isolation, and wrong judgment. Also, visual representations of symptoms of HIV/AIDS (e.g., severe weight loss) presented in the early 1980s during awareness creation has played a role on the intuition of the public about HIV as a deadly medical condition [22]. Some of these initial images connected to an HIV-positive diagnosis have unintendedly contributed to a lingering and heightened fear of contracting HIV and discouraged the uptake of HIV testing. Studies have shown that visual representations (i.e., in various media forms such as banners, television commercials, and films) of individuals affected with HIV/AIDS have different implications. For instance, some of these portrayals served to raise awareness about the disease’s impact and advocated for the rights of those living with HIV/AIDS, notably exemplified by the strategic use of visual imagery by activist groups like the Coalition to Unleash Power (ACT UP) from the early 1980s [23]. Conversely, other visual depictions prevalent in newspapers during the same period which aimed to instil ‘fear’ as a deterrent for people engaging in high-risk behaviors also worked to create and entrench the notion that some structurally exposed groups, such as sex workers, people who inject drugs (PWIDs) and men who had sex with other men (MSM) were those at risk of HIV/AIDS infection [23]. Thus, while accurate HIV knowledge dissemination is key to dispelling misconceptions about the disease and promoting the social inclusivity of PLHIV, such educational programs must be planned and executed in ways that do not reinforce the fear of HIV contraction among the public.

In the Ghanaian context, where HIV prevalence remains high and has been increasing in recent times (i.e., about 16,574 new cases and 9,359 deaths in 2022 alone), PLHIV face additional challenges living their usual lives in their respective communities due to misconceptions about the etiology of HIV and deeply ingrained ethno-cultural, social, and religious beliefs about its spread [24]. HIV misconceptions, including the belief that HIV is a deadly and incurable disease and principally caused by promiscuous sexual behaviors or the moral failings of individuals, drive the discrimination and social exclusion of PLHIV [24]. Available studies from Ghana have explored the lived experiences of PLHIV [7, 25–28]. According to Adam et al. [7] for instance, PLHIV in the Volta region of Ghana face stigma in the form of gossip and verbal harassment which induces non-disclosure of HIV status for fear of rejection and shame. Another study by Mumin et al. [29] examined the connections between the lived experiences of HIV seropositive people in urban Ghana, internalized stigma, and the impacts on efforts to combat the disease. While much work has focused on the lived experiences of PLHIV in Ghana, very little is known about the factors associated with HIV stigma endorsement in the country [11, 24, 29]. Thus, guided by the theory of planned behavior, this study contributes to the broader literature by exploring the factors that are influencing HIV-related stigma in Ghana.

### Theoretical context

The theory of planned behavior (TPB), propounded by Ajzen [30], is one of the most robust frameworks for explaining health-related behaviors [31]. TPB theorizes that three factors—*subjective norms (i*.*e*., the perceived social pressure to perform or not to perform the behavior*)*, *attitude towards the behavior* (*i*.*e*., the extent to which the individual has a favorable or unfavorable judgement of the act or behavior in question), *and perceived behavioral control* (*i*.*e*., the perceived ease or difficulty of performing the given behavior, and the presence of factors that may facilitate or hinder it.) interact to shape the behavioral intentions of an individual and influence the individual’s willingness to accept an idea or perform a given behavior.

“Intentions are assumed to capture the motivational factors that influence a behavior; they are indications of how hard people are willing to try, of how much of an effort they are planning to exert, in order to perform the behavior” [30]. However, behavioral intentions can only translate into actual behavior if the behavior in question (e.g., endorsement of HIV stigma) is under volitional control (i.e., only if the person can decide at will to discriminate or to not discriminate against PLHIV), according to TPB [30, 32]. Moreover, the performance and success of most health behaviors depend on nonmotivational factors such as the availability of necessary opportunities and resources, including knowledge and awareness, time, money, skills etc. [25]. For instance, one’s level of knowledge about HIV, antiretroviral therapy (ART), PrEP (pre-exposure prophylaxis), and the perceived risk of contracting HIV, may collectively influence decisions regarding the discrimination of PLHIV. Thus, with the required opportunities and resources (e.g., access to accurate HIV information) and intent to perform the behavior (e.g., create an inclusive environment for PLHIV), the propensity of success is high (i.e., the likelihood of rejecting the endorsement of stigma and discrimination of PLHIV), holding all other things constant.

**Fig 1:**
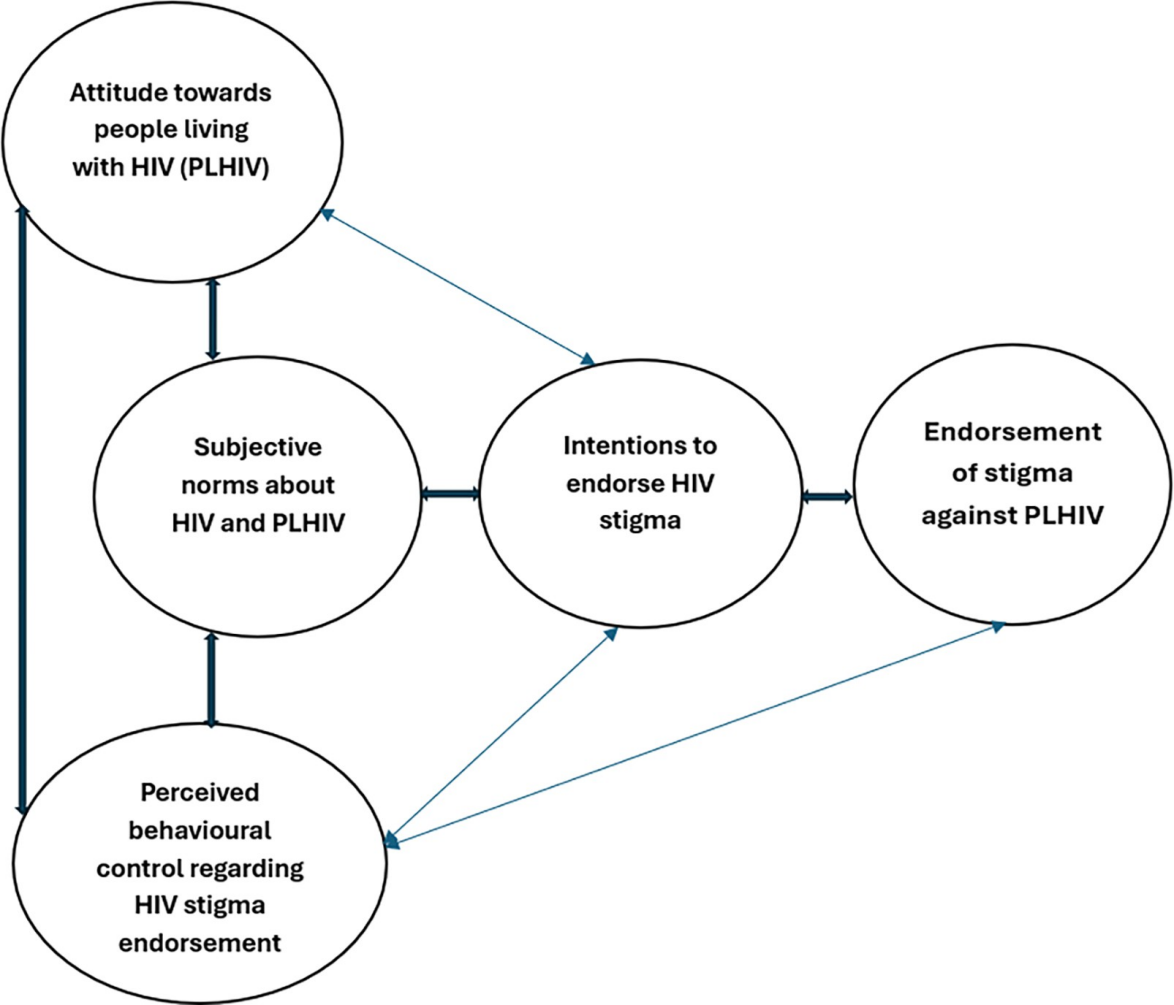
A modified diagrammatic representation of TPB in the context of HIV stigma endorsement. Source: [30].

**TPB** emphasizes the need to understand that these factors (i.e., subjective norms, attitudes, and perceived behavioral control) are not deterministic but, rather provide a framework for understanding the underlying factors influencing intents and behaviors. TPB has been widely adopted and utilized in the field of health sciences to study health-related behaviors [31, 32] with *attitude toward the action and perceived behavioral control* as significant variables accounting for possible variation in intentions behind the behavior in question [33]. Thus, adopting TPB, this study explored the factors influencing the endorsement of stigma against PLHIV in Ghana. Fig 1 below is a modified diagrammatic representation of TPB within the context of HIV stigma endorsement.

## Methods

### Data collection

This study draws data from the 2022 Ghana Demographic and Health Survey (GDHS), implemented by the Ghana Statistical Service (GSS) [18]. Data collection took place from 17 October 2022 to 14 January 2023. Technical assistance to the Demographic and Health Surveys (DHS) Program was provided by the International Coaching Federation (ICF). To ensure that the survey procedures were in accordance with U.S. and international ethical research standards, ICF submitted the GDHS survey protocol to the ICF Institutional Review Board (IRB) for ethical clearance. Ethical clearance for the survey was granted by the IRB. Prior to data collection, officials of GSS obtained verbal informed consent from all eligible participants, and responses of these consents were recorded in the survey questionnaire. For details on informed consent, visit: https://dhsprogram.com/pubs/pdf/FR387/FR387.pdf.

### Sampling design and frame

For the 2022 GDHS, a stratified representative sample of 18,450 households were selected in 618 clusters, which resulted in a total sample size of 22,058 (7,044 interviewed men and 15,014 interviewed women aged 15–49 and 15–59 in one of every two households selected). The sampling frame for the 2022 GDHS is an updated version of the 2021 Population and Housing Census, prepared by the GSS. The sampling procedure, a stratified two-stage cluster sampling, was designed to yield representative results at the national level, for urban (localities with population of 5,000 or more) and rural areas (localities with a population of less than 5,000) [34] and for each of the country’s 16 regions for most DHS indicators. In the first stage, 618 target clusters were selected from the sampling frame using a probability proportional to size strategy for urban and rural areas in each region. In the second stage, after the selection of clusters, a household listing and map operation was carried out in all the selected clusters to develop a list of households for each cluster. This list then served as a sampling frame for the selection of the household sample. Prior to data collection, GSS performed a pretest of the survey questionnaire and staff training. Also, the informed consent of the participants was sorted and obtained before conducting the survey [18].

### Measures

Outcome variables: Two outcome variables were evaluated for HIV stigma endorsement. Thus, respondents were questioned: (1) “Would you buy fresh vegetables from a shopkeeper or vendor if you knew that this person had HIV?”, and (2) “Do you think children living with HIV should be allowed to attend school with children who do not have HIV?”. For these questions, a “no” response meant an endorsement of a stigmatizing attitude, according to the Ghana Statistical Service [18] description of indicators of stigma and discrimination against PLHIV.

Explanatory variables: Knowledge of HIV, including whether one can get HIV by sharing food with PLHIV, whether a healthy-looking person can have HIV, and whether respondents are aware of drugs to help HIV infected people live longer. Also, the educational attainment of respondents, their ages, marital status, household wealth, religious affiliation, type of place of residence (Urban vs. Rural), and the region of residence were accounted for. Household wealth was derived using the Principal Component Analysis (PCA) technique. Following the PCA technique, households are assigned scores based on the number and kinds of goods or properties they own, ranging from a television to a bicycle or car, and housing characteristics such as source of drinking water, toilet facilities, and flooring materials. National wealth quintiles are compiled by assigning the household score to each usual (de jure) household member, ranking each person in the household population by his or her score, and then dividing the distribution into five equal categories, each comprising 20% of the population [18].

The selection of variables for our bivariate and multivariate analysis was informed by both the theory (i.e., theory of planned behaviour) of the study, as well as a review of the existing literature. Notably, only three of our explanatory variables have missing values for which we employed the Complete Case Analysis (CCA) approach. While acknowledging that CCA may imply a loss of information on the missing data points, it was, however, employed to preserve the original relationships between variables in the dataset. Besides, given that this paper focuses on identifying the factors associated with HIV stigma endorsement, combining missing values with any of the original response categories may nullify the validity and implications of the study’s findings.

### Analytical approach

Both descriptive and inferential statistical analyses have been employed in this study. First, a statistical distribution table has been used to present socio-demographic characteristics of the study sample. Also, logistic regression models were used to examine the relationship between each explanatory variable and HIV stigma endorsement. Both binary and multiple logistic regression analyses were conducted to ascertain the factors associated with HIV stigma endorsement in the study context. All statistical data analyses were performed in Stata version 18.

## Results

### Sample characteristics

Results for the sample characteristics are presented in Table 1. A majority (56.80%) of the respondents, with their ages ranging from 15–59 years, had secondary education. Also, the majority (52.23%) were married/living with a partner and belonged to the Christian (61.73%) religious faith. Surprisingly, most respondents (56.48) were against allowing children living with HIV to attend school. Likewise, despite majority (68.94%) being aware that HIV cannot be acquired through the sharing of food, they nonetheless expressed disapproval of buying food from vendors with HIV (66.79%). See Table 1 for details on the sample characteristics.

**Table 1:** Descriptive statistics of study sample.

Variable	Frequencies (%)
**Education**	
No education	1186 (16.84)
Primary	923 (13.10)
Secondary	4001 (56.80)
Higher	934 (13.26)
**Age**	Mean (32), SD (12.2), Min (15), Max (59), Median (37), IQR (17)
**Wealth**	
Poorest	1849 (26.25)
Poorer	1553 (22.05)
Middle	1318 (18.71)
Richer	1235 (17.53)
Richest	1089 (15.46)
**Marital status**	
Never in a union	3047 (43.26)
married/living with partner	3679 (52.23)
Widowed/divorced/separated	318 (4.51)
**Religion**	
Christianity	4348 (61.73)
Islam	1993 (28.29)
Traditionalist	348 (4.94)
No religion/other	355 (5.04)
**Ethnicity**	
Akan	2412 (34.24)
Ga/Dangme	334 (4.74)
Ewe	765 (10.86)
Guan	369 (5.24)
Mole-Dagbani	1811 (25.71)
Grusi	352 (5.00)
Gurma	667 (9.47)
Mande	236 (3.35)
Other	98 (1.39)
**Region**	
Western	382 (5.42)
Central	442 (6.27)
Greater Accra	493 (7.00)
Volta	335 (4.76)
Eastern	389 (5.52)
Ashanti	491 (6.97)
western north	402 (5.71)
Ahafo	422 (5.99)
Bono	351 (4.98)
Bono east	500 (7.10)
Oti	467 (6.63)
Northern	531 (7.54)
Savannah	548 (7.78)
North East	416 (5.91)
Upper East	464 (6.59)
Upper West	411 (5.83)
**Type of place of residence**	
Urban	3251 (46.15)
Rural	3793 (53.85)
**Can get HIV by sharing food with PLHIV**	
No	1574 (68.94)
Yes	668 (29.26)
Don’t know	41 (1.80)
**Healthy looking person can have HIV**	
No	653 (28.60)
Yes	1594 (69.82)
Don’t know	36 (1.58)
**Heard of drugs to help HIV infected people live longer**	
No	2420 (35.84)
Yes	4333 (64.16)
**Ever been tested for HIV**	
No	5298 (75.21)
Yes	1746 (24.79)

Min = Minimum, Max = Maximum, SD = Standard Deviation, IQR = Interquartile Range.

#### Bivariate analysis of stigma endorsement against PLHIV

Results for bivariate analysis are presented in Table 2. From the results, participants with the misconception that HIV can be transmitted through sharing of food reported higher odds of HIV stigma endorsement against children living with HIV (OR = 4.235; P<0.001) and vendors with HIV (OR = 3.627; P<0.001) when compared to those without such misconception. On the other hand, knowing that a healthy-looking person can have HIV, was significantly associated with lower odds of stigma endorsement against children living with HIV (OR = 0.415; P<0.001) and vendors with HIV (OR = 0.429; P<0.001). Likewise, having knowledge of drugs that help HIV infected people live longer, and ever being tested for HIV, were significantly associated with lower odds of HIV stigma endorsement (see Table 2). Also, educational attainment, age, wealth, religious affiliation, ethnicity, region and type of place of residence (urban vs. rural) significantly predicted HIV stigma endorsement at varied levels (see Table 2).

**Table 2:** Bivariate regression results estimating stigma endorsement against PLHIV.

VARIABLES	Children living with HIV/AIDS		Vendors living with HIV/AIDS	
	OR (SE)	I95%CI	OR (SE)	I95%CI
**Can get HIV by sharing food with PLHIV** (Ref: no)				
Yes	4.235(0.497) ***	3.364 5.331	3.627(0.469) ***	2.815 4.675
Don’t know	1.384(0.454)	0.727 2.634		
**Healthy looking person can have HIV** (Ref: no)				
Yes	0.415(0.044) ***	0.337 0.511	0.429(0.050) ***	0.340 0.541
Don’t know	1.034(0.425)	0.461 2.318	0.820(0.356)	0.350 1.922
**Heard of drugs to help HIV infected people live longer** (Ref: no)				
Yes	0.429(0.023) ***	0.387 0.477	0.423(0.024) ***	0.377 0.474
**Ever been tested for HIV** (Ref: No)				
Yes	0.370(0.021) ***	0.330 0.414	0.374(0.021) ***	0.334 0.418
**Education** (ref: No education)				
Primary	0.698(0.075) ***	0.565 0.863	0.647(0.083) ***	0.503 0.833
Secondary	0.332(0.027) ***	0.283 0.390	0.288(0.028) ***	0.237 0.350
Higher	0.072(0.007) ***	0.058 0.089	0.066(0.007) ***	0.052 0.083
**Age**	0.985(0.002) ***	0.981 0.989	0.988(0.002) ***	0.984 0.992
**Marital status** (Ref: Never in a union)				
married/living with partner	0.915(0.046)	0.829 1.011	0.910(0.048)	0.820 1.011
Widowed/divorced/separated	0.795(0.094)	0.629 1.003	0.907(0.113)	0.709 1.159
**Wealth** (Ref: Poorest)				
Poorer	0.615(0.047) ***	0.529 0.714	0.579(0.050) ***	0.488 0.686
Middle	0.452(0.035) ***	0.387 0.527	0.409(0.035) ***	0.345 0.486
Richer	0.367(0.029) ***	0.314 0.429	0.302(0.026) ***	0.254 0.358
Richest	0.204(0.017) ***	0.173 0.241	0.164(0.014) ***	0.137 0.195
**Religion** (Ref: Christianity)				
Islam	1.510(0.085) **	1.351 1.688	1.452(0.087) **	1.290 1.634
Traditionalist/spiritualist	2.353(0.298) ***	1.836 3.017	2.446(0.351) ***	1.84 3.243
No religion/other	1.834(0.223) ***	1.444 2.329	1.688(0.221) ***	1.305 2.183
**Ethnicity** (Ref: Akan)				
Ga/Dangme	0.895(0.105)	0.711 1.128	1.033(0.126)	0.813 1.312
Ewe	0.953(0.080)	0.808 1.123	0.866(0.074)	0.732 1.024
Guan	0.972(0.110)	0.778 1.213	1.129(0.134)	0.894 1.425
Mole-dagbani	1.207(0.077) **	1.064 1.368	1.325(0.089) ***	1.161 1.512
Grusi	0.837(0.098)	0.664 1.055	1.568(0.205) ***	1.214 2.027
Gurma	3.414(0.367) ***	2.765 4.217	3.012(0.350) ***	2.398 3.784
Mande	1.303(0.194)	0.972 1.747	1.299(0.205)	0.953 1.770
Other	1.047(0.223)	0.689 1.590	1.226(0.277)	0.786 1.911
**Region of residence** (Ref: Western)				
Central	0.725(0.105) *	0.544 0.965	1.026(0.154)	0.764 1.377
Greater Accra	0.527(0.074) ***	0.400 0.695	0.686(0.097) **	0.519 0.907
Volta	0.454(0.069) ***	0.335 0.614	0.585(0.090) ***	0.432 0.793
Eastern	0.471(0.070) ***	0.352 0.631	0.767(0.115)	0.571 1.030
Ashanti	0.547(0.077) ***	0.415 0.722	0.834(0.119)	0.629 1.105
western north	0.582(0.085) ***	0.436 0.777	0.857(0.128)	0.638 1.151
Ahafo	0.594(0.086) ***	0.446 0.790	0.972(0.145)	0.724 1.305
Bono	0.430(0.065) ***	0.318 0.580	0.776(0.120)	0.572 1.052
Bono east	0.768(0.109)	0.580 1.016	1.156(0.171)	0.865 1.546
Oti	1.201(0.177)	0.899 1.605	1.257(0.188)	0.937 1.688
Northern	1.375(0.204) *	1.028 1.839	2.148(0.342) ***	1.572 2.936
Savannah	0.892(0.128)	0.672 1.184	1.847(0.290) ***	1.358 2.514
North east	1.795(0.290) ***	1.307 2.465	2.002(0.334) ***	1.443 2.778
Upper East	0.408(0.058) ***	0.308 0.541	0.635(0.091) **	0.478 0.843
Upper West	0.548(0.080) ***	0.410 0.731	1.220(0.189)	0.900 1.654
**Type of place of residence** (Ref: Urbana)				
Rural	1.731(0.085) ***	1.570 1.907	1.985(0.104) ***	1.791 2.200

*P<0.05

**P<0.01

***P<0.001; Odd Ratio (OR), Standard Error (SE), Confidence Interval (CI)

### Multivariate analysis of stigma endorsement against PLHIV

The results for multivariate analysis are also presented in Table 3. Expectedly, and consistent with results at the bivariate level, the notion that HIV can be transmitted through sharing of food with PLHIV, was significantly associated with a higher likelihood of stigma endorsement against children with HIV (OR = 3.381; P<0.001), and vendors with HIV (OR = 3.00; P<0.001). On the contrary, knowing that a healthy-looking person can be living with HIV, was significantly less associated with the endorsement of stigma against children living with HIV (OR = 0.505; P<0.001), and vendors living with HIV (OR = 0.573; P<0.001). Likewise, having knowledge of drugs that help HIV infected people to live longer was significantly less associated with endorsement of stigma against children living with HIV (OR = 0.768; P<0.01), and vendors living with HIV (OR = 0.719; P<0.001). Moreover, participants with higher levels of educational attainment were significantly less associated with endorsement of stigma against children living with HIV (OR = 0.255; P<0.01), and vendors with HIV (OR = 0.327; P<0.01). Also, age was significantly and inversely associated with the endorsement of stigma against children living with HIV (OR = 0. 0.951; P<0.05), and vendors living with HIV (OR = 0.961; P<0.05). Further, wealth, religious affiliation, ethnicity, and the region of residence significantly predicted HIV stigma endorsement in the study context (see Table 3).

**Table 3:** Multiple regression results estimating stigma endorsement against PLHIV.

VARIABLES	Children living with HIV/AIDS		Vendors living with HIV/AIDS	
	OR (SE)	95% CI	OR (SE)	95% CI
**Can get HIV by sharing food with PLHIV** (Ref: no)				
Yes	3.381(0.425) ***	2.642 4.326	3.00(0.415) ***	2.291 3.938
Don’t know	0.944(0.368)	0.439 2.030	2.775(01.360) *	1.061 7.252
**Healthy looking person can have HIV** (Ref: no)				
Yes	0.505(0.059) ***	0.400 0.637	0.573(0.073) ***	0.445 0.738
Don’t know	1.100(0.513)	0.441 2.745	0.503(0.246)	0.193 1.315
**Heard of drugs to help HIV infected people live longer** (Ref: no)				
Yes	0.768(0.079) *	0.627 0.941	0.719(0.078) **	0.580 0.890
**Ever been tested for HIV** (Ref: No)				
Yes	0.866(0.149)	0.617 1.214	0.772(0.132)	0.551 1.082
**Education** (ref: No education)				
Primary	0.584(0.190)	0.308 1.107	1.326(0.433)	0.699 2.515
Secondary	0.375(0.115) ***	0.205 0.684	0.760(0.226)	0.424 1.363
Higher	0.255(0.095) ***	0.122 0.529	0.327(0.118) ***	0.160 0.667
**Age** (Ref: 15–19)	0.951(0.018) *	0.915 0.988	0.961(0.019) *	0.923 1.000
**Marital status** (Ref: Niver in union)				
married/living with partner	1.294(0.278)	0.849 1.972	1.031(0.228)	0.667 1.593
Widowed/divorced/separated	3.342(01.988)	1.041 10.724	1.390(0.767)	0.471 4.101
**Wealth** (Ref: Poorest)				
Poorer	0.716(0.111) *	0.528 0.973	0.701(0.117) *	0.504 0.974
Middle	0.790(0.143)	0.553 1.128	0.732(0.142)	0.500 1.071
Richer	0.600(0.119) *	0.406 0.886	0.564(0.119) **	0.372 0.854
Richest	0.460(0.103) ***	0.296 0.715	0.460(0.108) ***	0.290 0.730
**Religion** (Ref: Christianity)				
Islam	1.417(0.218)	1.047 1.917	1.054(0.174)	0.763 1.457
Traditionalist/spiritualists	1.295(0.433)	0.672 2.495	1.450(0.532)	0.706 2.978
No religion/other	0.726(0.196)	0.426 1.234	0.570(0.155)	0.334 0.972
**Ethnicity** (Ref: Akan)				
Ga/Dangme	0.726(0.187)	0.437 1.205	0.705(0.189)	0.416 1.194
Ewe	0.968(0.218)	0.622 1.505	0.723(0.168)	0.458 1.140
Guan	0.712(0.185)	0.428 1.185	0.794(0.219)	0.462 1.366
Mole-Dagbani	0.668(0.124) *	0.464 0.961	0.701(0.138)	0.476 1.034
Grusi	0.450(0.116) **	0.270 0.747	1.012(0.294)	0.572 1.790
Gurma	1.180(0.293)	0.725 1.921	1.210(0.318)	0.722 2.026
Mande	0.494(0.168) **	0.253 0.964	0.506(0.174) *	0.257 0.995
Other	0.709(0.304)	0.306 1.643	1.157(0.580)	0.433 3.092
**Region of residence** (Ref: Western)				
Central	0.619(0.170)	0.360 1.062	0.723(0.207)	0.412 1.267
Greater Accra	0.536(0.157) *	0.302 0.952	1.083(0.334)	0.591 1.983
Volta	0.387(0.136) **	0.193 0.772	0.564(0.204)	0.277 1.149
Eastern	0.354(0.102) ***	0.201 0.623	0.472(0.141) *	0.263 0.848
Ashanti	0.374(0.097) ***	0.224 0.624	0.533(0.144) *	0.314 0.905
western north	464(0.133) **	0.264 0.817	0.536(0.159) *	0.299 0.959
Ahafo	0.413(0.121) **	.0231 0.735	0.480(0.146) *	0.264 0.873
Bono	0.4401(0.131) **	0.244 .791	0.581(0.180)	0.316 1.069
Bono east	0.416(0.123) **	0.232 0.745	0.622(0.194)	0.337 1.149
Oti	0.551(0.177)	0.292 1.036	0.436(0.142) *	0.229 0.828
Northern	0.652(0.222)	0.334 1.273	0.861(0.306)	0.429 1.729
Savannah	0.378(0.121) **	0.201 0.711	0.749(0.260)	0.379 1.481
North East	0.519(0.180)	0.262 1.027	0.888(0.330)	0.429 1.840
Upper East	0.265(0.082) ***	0.144 0.486	0.478(0.153) *	0.255 0.895
Upper West	0.311(0.099) ***	0.167 0.582	0.866(0.302)	0.437 1.716
**Type of place of residence** (Ref: Urbana)				
Rural	1.180(0.144)	0.928 1.501	1.006(0.130)	0.780 1.296

*P<0.05

**P<0.01

***P<0.001; Odd Ratio (OR), Standard Error (SE), Confidence Interval (CI)

## Discussion

Despite the progress made in reducing new HIV infections in Ghana over the last decade, evidence suggests the endorsement of HIV stigma and discrimination may be driving new HIV infections in the country by working to discourage HIV testing and the uptake of HIV treatment for PLHIV. Given that the endorsement of HIV-related stigma and discrimination has been found to be gender-specific in the context of Ghana, this study, as part of a bigger research project, examined the factors associated with HIV stigma endorsement among men in Ghana. Guided by the Theory of Planned Behavior, our results indicated that participants awareness about HIV being asymptomatic, having knowledge of drugs that help PLHIV to live longer, and higher levels of educational attainment were significantly less associated with the endorsement of HIV stigma. On the contrary, misconception about HIV transmission (i.e., through sharing of food with PLHIV) was significantly associated with a higher likelihood of HIV stigma endorsement among men in Ghana. Furthermore, age, wealth, religious affiliation, ethnicity, and the region of residence significantly predicted HIV stigma endorsement in the study context, in different directions. The observed significant association between HIV misconception (i.e., HIV can be transmitted through the sharing of food) and the higher the likelihood of stigma endorsement against PLHIV underscores the urgent need to intensify public awareness of the etiology and spread of HIV in the context of Ghana if the country is to achieve the UNAIDS 95-95-95 target by 2030 [17]. HIV misconceptions work to induce social exclusion and discrimination of PLHIV. Prior studies in similar contexts have highlighted the debilitating role of HIV misconceptions in the endorsement of HIV-related stigma [19, 35]. On the other hand, having HIV knowledge, that is, knowing that HIV can be asymptomatic (i.e., a healthy-looking person can have HIV), and being aware of medication that prolongs the life of PLHIV, were significantly less associated with HIV stigma endorsement, a finding that reaffirms the of theory planned behaviour’s postulation regarding the performance and success of most behaviors being dependent on nonmotivational factors such as knowledge. People with adequate knowledge of ART may also be positioned to understand that HIV is treatable and cannot be spread through mere interactions or sharing of food with an infected person, thus making them more receptive to PLHIV. Across SSA, studies suggest that the availability of ART has normalized HIV as a chronic health condition [36], although others indicate a rise in stigma despite increased ART availability [37]. Treves-Kagan et al. [38] in their community-based study on ART availability and HIV-related stigma have confirmed the coexistence of both realities. They, however, emphasized that although negative attitudes towards PLHIV persist, the presence of ART has undoubtedly worked to reduce the perceived deadliness of the virus and other related misconceptions (e.g., HIV being an incurable disease) [38]. While it is important to emphasize that knowledge of ART may not automatically reduce the endorsement of HIV-related stigma, studies have pointed to the positive impact of ART on lower levels of HIV stigma [38, 39]. Therefore, as part of a holistic approach to eliminate HIV-related stigma in Ghana, and to promote the social inclusivity of PLHIV, more coordinated efforts by important stakeholders (particularly, Ghana AIDS Commission), are needed for awareness creation on important topics such as ART, PrEP, Post-Exposure Prophylaxis (PEP), and U = U (Undetectable = Untransmissible) among others. Intensification of accurate HIV/AIDS knowledge dissemination has the potential of drastically reducing HIV misconceptions and stigma towards PLHIV. Also, a unit increase in age was associated with decreased odds of HIV stigma endorsement in our study context, a finding that can be explained by some intersecting factors, including one’s level of education and HIV awareness as well as socioeconomic status. For instance, an earlier study in Ghana shows that people with higher educational attainment have more exposure to media information and public health programs on HIV, which tend to broaden their knowledge and understanding of the disease [20]. A longitudinal study by Srivastava et al. [40] further suggests that level of educational attainment is a mediator for an observed positive association between age and knowledge of HIV/AIDS. While exploring misconceptions and HIV stigma in Indonesia, Suantari [19] also uncovered that lower level of educational attainment was more associated with misconceptions about HIV, which is a major driver of HIV-related stigma. Thus, given that a unit increase in age is significantly associated with lower odds of HIV stigma endorsement, the youngsters must be targeted for HIV knowledge dissemination and stigma reduction programs in Ghana. We further observed that, consistent with earlier studies [5, 41, 42], higher level of educational attainment was significantly associated with lower odds of endorsing HIV stigma which highlights the crucial role of formal education in understanding the etiology and spread of diseases, including HIV. In a Ugandan study, it was established that education had a causal effect of significantly reducing HIV-related stigma [5]. The positive impact of formal education on health information access and health-related behaviour has been well documented [43–47]. For example, a recent study by Antabe and Sano [9] in Haiti showed that people with higher educational attainment were less associated with the endorsement of HIV misconceptions, a finding that reaffirms the positive impact of higher education on individuals’ knowledge of important health topics, as well as their behaviours. Therefore, the possibility of people with formal education having higher levels of health literacy may account for their observed lower odds of HIV stigma endorsement in our study. To address disparities in HIV information access, HIV awareness and stigma reduction programs must be carried out both in English language and local dialects to accommodate people without formal educational attainment.

Moreover, compared to members of the poorest households, those from relatively wealthy households reported lower odds of HIV stigma endorsement. Similar findings have been established in Ghana, where Tenkorang and Owusu [24] indicated that people from wealthy households are less likely to discriminate or stigmatize PLHIV relative to those from poorer households. Other studies suggest that wealthier people have more exposure to knowledge and awareness about HIV transmission, and, therefore less likely to hold misconceptions about the disease [48]. Socioeconomic status often shapes different levels of vulnerabilities and privileges in society, particularly, health information access [49]. For instance, people from weaker socioeconomic backgrounds may have limited exposure and access to health information resources (including internet, television, radio etc.) relative to those from better socio-economic backgrounds [50]. Such disparities in health information access may have detrimental health implications, including widespread misconceptions about HIV and discrimination against PLHIV. Therefore, HIV awareness programs and stigma reduction efforts must target people from poorer socioeconomic backgrounds, particularly, the uneducated.

Furthermore, ethnicity significantly predicted HIV stigma endorsement, where participants from the Mande ethnic background reported lower odds of HIV stigma endorsement compared to those from the Akan ethnic background. This finding may point to the role of cultural perceptions, interpretations and understanding about the etiology of HIV. Where this cultural and ethnic interpretation largely deviates from the clinical explanation for the spread of HIV, members of the group tend to hold stigmatizing attitudes, especially if the symptoms of HIV/AIDS have some similarities with existing diseases for which there is an entrenched cultural belief about its spread [51]. In Malawi, a similar finding has been made by Antabe et al. [52], who stated that the description of all sexually transmitted infections in the Chewa language predisposed members of this ethnic group who are the majority in the Central region of the country to the endorsement of HIV misconception and discrimination. While investigation the drivers of myths and HIV misconceptions in Ghana, Tenkorang [53] noted that, compared to respondents from other ethnic groups, the Akans were more likely to endorse myths about HIV, and such misconceptions have been widely associated with HIV stigma endorsement [19, 35]. Specifically in Ghana, in order to devise and implement effective strategies for stigma reduction, HIV education programs and policies must consider, or understand the ethnic beliefs of communities regarding HIV.

Geographical variations were further observed in HIV-related stigma endorsement in the study context. For instance, compared to participants from the Western region, those from the Upper East, Upper West, Eastern, Ashanti, Western North, and Ahafo were significantly less associated with `HIV stigma endorsement. Inferring from the national HIV prevalence rates, the rising rates of HIV prevalence in some regions may be instilling a heightened fear of the spread of the virus, hence, the increased tendency of people in those regions to endorse HIV stigma. Evidence from the Ghana AIDS Commission suggests that, with the exception of Ashanti region (76,672 PLHIV) and Eastern region (42,446 PLHIV), the estimated number of PLHIV in Western region (19,731 PLHIV) is higher than the Upper East (5,776 PLHIV), Upper West (3,657 PLHIV), Western North (11,331 PLHIV), and Ahafo (7,068 PLHIV) [54]. Earlier studies in Ghana suggest that regions with relatively high HIV prevalence were more likely to endorse myths about HIV [53]. Meanwhile, misconceptions about HIV drives the endorsement of HIV-related stigma and discrimination [19, 35]. Also, within each region, the size of communities may play a role in HIV-related stigma or discrimination. For instance, inhabitants of smaller communities are close-knitted, making privacy related to an HIV-positive diagnosis relatively challenging compared to urban centres [55]. All these contextual factors must be incorporated into the formulation of HIV stigma reduction policies and programs in Ghana.

There are some noteworthy limitations of this study. First, given the quantitative approach to this study, the interpretation of the results is only limited to statistical association. Thus, future research should consider integrating qualitative approaches where researchers can unpack the depth of the factors influencing the endorsement of HIV stigma and discrimination among men in Ghana. Also, given the sensitive nature of the topic (i.e., discrimination against PLHIV) in the context of Ghana, this can potentially introduce bias in the response of study participants. That notwithstanding, the findings in this study are relevant for targeting men as a key sub-population group for HIV education and sensitization.

## Conclusions

Despite the above limitations, findings from this study have some valuable policy implications. First and foremost, there is the urgent need for the increased targeting of men in Ghana with accurate HIV information, given the observed significant association between HIV misconception and the higher odds of discrimination against PLHIV. Specifically, educational programs emphasizing that HIV cannot be spread through casual contact (e.g., sharing common spaces like classrooms, or food with PLHIV) are crucial for dispelling misconceptions, reducing discrimination, and promoting the social inclusion of PLHIV. Such initiatives can be integrated into the school curricula, community outreach programs, public health campaigns, faith-based organizations, and media outlets. Secondly, the study identifies demographic factors such as age, wealth, educational attainment, and region of residence as determinants of HIV stigma endorsement. Therefore, policymakers should consider developing targeted interventions that address the unique challenges and perspectives of varying demographics on the topic of HIV stigma. A collaboration between relevant stakeholders including health authorities, educational institutions, community organizations, policymakers, and PLHIV is a prerequisite for the implementation of HIV stigma reduction initiatives. Reduction in the discrimination and stigmatization of PLHIV is also a prerequisite for promoting testing, status disclosure, and treatment uptake or adherence to treatment.

Knowledge of HIV/AIDS, level of education, age, wealth, ethnic beliefs about the etiology of HIV/AIDS, and geographical location are all factors associated with the endorsement of HIV-related stigma in Ghana. By improving access to accurate information on HIV, and by promoting the social inclusion of PLHIV, Ghana can significantly reduce HIV-related stigma and discrimination, all of which will foster testing, status disclosure and treatment uptake.
